# A visual search asymmetry for plaids

**DOI:** 10.1177/03010066251340285

**Published:** 2025-05-30

**Authors:** Joshua A Solomon, Michael J Morgan, Charles F Chubb

**Affiliations:** School of Health & Medical Sciences, City St George's, University of London, UK; School of Health & Medical Sciences, City St George's, University of London, UK; University of California, Irvine, California, United States

**Keywords:** visual search, attentional capture, segmentation, texture

## Abstract

Search asymmetry has been called a “litmus test” for basic visual features. The letter Q is thought to contain a basic feature because (*i*) it can be found quickly, no matter how many O's it is hiding amongst and (*ii*) it is much harder to find an O amongst Q's. We tested the possibility that a basic visual feature is created when two perpendicular Gabor patterns are superimposed to form a “plaid.” We found relatively large effects of set size on reaction time whenever participants tried to find a Gabor hiding among plaids. Set-size effects were smaller when participants tried to find a 2- or 4-cycle-per-degree plaid that was hiding among its component Gabors. The implication is that these plaids contain a basic visual feature, which is not present in its component Gabors. This feature may be an intrinsic two-dimensionality that is extracted from the visual intensity map. Mixed-frequency plaids did not pop out from their component Gabors. This last result suggests that the visual system separates intrinsically two-dimensional image regions (e.g., corners and junctions) from intrinsically one-dimensional image regions (e.g., straight edges) after the scene is segregated into parallel spatial frequency channels.

## Introduction

Nam et al. (2009) inferred the existence of a preattentive mechanism (a “plaid grabber”) responsible for detecting the superimposition of perpendicular but otherwise identical gratings from the results of a visual search experiment in which participants had to discriminate sets of 4 or 8 gratings from sets of 3 or 7 of these gratings (“distractors”) plus one plaid (the “target”). When all gratings and both plaid components had the same spatial frequency, the plaid “popped out,” that is, there was little effect of set size on response time. Larger effects of set size were found when distractors and targets contained gratings of different spatial frequency.

Certainly, some variety of preattentive processing seems necessary to explain pop out. However, whereas pop out is typically considered a necessary property for targets that contain a feature capable of attracting attention, it isn’t typically considered sufficient (Wolfe & Horowitz, 2004). Another necessary property is search asymmetry, that is, a failure of pop out when target and distractor identities are switched (Treisman & Souther, 1985). Consequently, we thought it prudent to check for search asymmetry before wholly endorsing the existence of plaid grabbers within the preattentive visual system.

## Methods

Although our methods were informed by those of Nam et al. (2009), we were keen to use the larger set sizes with which asymmetries have been reported (e.g., Wertheim et al. 2006). Another major difference between our methods is that all of our displays contained a target. The participant's task was to report whether this target was to the left or right of the vertical meridian bisecting the display. Aside from these differences, our methods were fairly similar to those of Nam et al.

The study was conducted at City University London in 2008. It adhered to the tenets of the Declaration of Helsinki. All four participants (including JAS) worked as visual psychophysicists in Solomon and Morgan's shared laboratory. Ages ranged from 25 to 45 years. None suffered from any visual pathology. The experiment was conducted on an iMac computer, running the PsychToolbox (Brainard, 1997; Pelli, 1997). Computer code has been included in the Supplemental Material.

All stimuli were composed of Gabor patterns. At the viewing distance of 0.57 m, each Gabor was the product of a sinusoidal luminance (“carrier”) grating having either 2 or 4 cycles per degree of visual angle^1^ and a circular Gaussian “window” having space constant *σ* = 0.31 degrees. The center of each Gaussian coincided with the carrier's transition from positive to negative Weber contrast. Isolated Gabors were displayed with contrasts that were independently selected from the uniform distribution over the interval (0.80, 1). Plaid components were displayed with contrasts that were independently selected from the uniform distribution whose minimum and maximum values were √2 lower (i.e., 0.57 and 0.71). Reasons for contrast randomization and the √2 relationship were described by Nam et al. (2009). The display was notionally partitioned into a 12 × 12 grid. On each trial, an equal number of positions on each side of the grid's vertical midline were occupied by either an individual Gabor or a plaid. Given this constraint, the specific position and orientation of each Gabor and plaid was selected at random.

**Figure 1.**
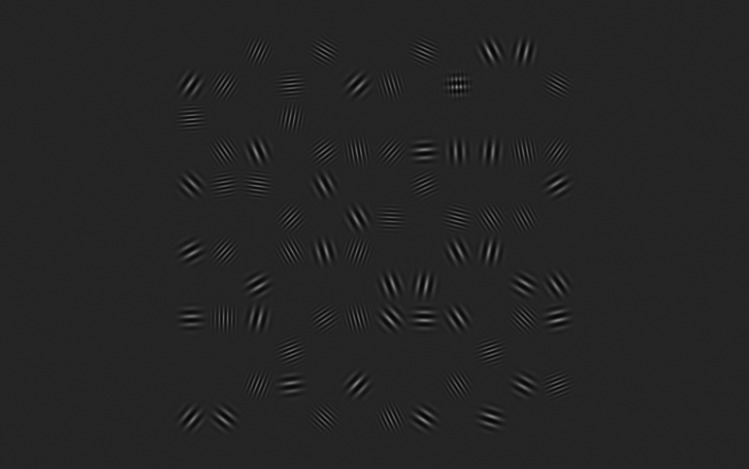
Screenshot of a dense display with a mixed-frequency target (upper right). Screenshots from the other 13 conditions have been included in the Supplemental Material.

On each trial the participant was required to indicate with a keypress whether the target was in one of the positions on the left side of the grid or one of the positions on the right side. They were instructed to respond as quickly and accurately as possible. All four participants completed two or three 50-trial blocks in each of 14 conditions, half of which featured “sparse” displays in which *N* = 18 grid positions were occupied and half of which featured “dense” displays in which *N* = 72 positions were occupied. In four conditions (two sparse, and two dense) all carriers had 2 cycles per degree. In half of these conditions, the target was a plaid, and the distractors were Gabors; in the other half the target was a Gabor, and the distractors were plaids. Another four conditions were similar, except that all carriers had 4 cycles per degree. In the remaining six conditions, each display contained nearly identical numbers of 2- and 4-cycle-per-degree carriers. In two of these conditions, the target was a mixed-frequency plaid, and the distractors were mixed-frequency Gabors (see Figure 1 for an example); in another two the target was a 2-cycle-per degree Gabor, and the distractors were mixed-frequency plaids; and in the final two the target was a 4-cycle-per degree Gabor, and the distractors were mixed-frequency plaids.

## Results

**Figure 2.**
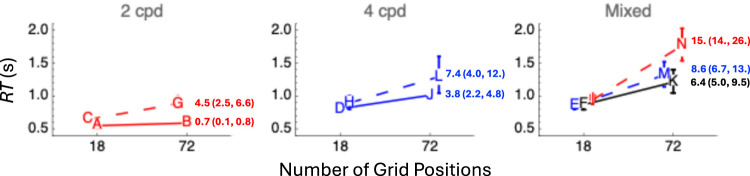
Geometric mean response times (*RT*) in all experimental conditions. Capital letters A–N index the fastest (A) through the slowest (N) of these mean *RT*s in alphabetical order. Letters connected by solid lines illustrate searches for a plaid amongst Gabors. Letters connected by dashed lines illustrate searches for a Gabor among plaids. Letters have been nudged laterally for legibility. Error bars contain two standard deviations (across four participants). The lower the ratio, the more efficient the search. The online version has color-coded numbers corresponding to the ratio Δ*RT*/Δ*N* in ms/item. Red, blue, and black indicate 2-cpd, 4-cpd, and mixed-frequency targets, respectively. Parentheses contain the range of ratios across participants (minimum and maximum).

Data from individual observers may be found in the Appendix. Figure 2 summarizes the response times from all trials with correct responses. Note that the searches for single-frequency plaids were more efficient than all other searches. Specifically, the difference between the geometric mean (weighted^2^) response time with dense displays and that for sparse displays was smallest when targets were either low-frequency plaids or high-frequency plaids. Note, however, that the search for mixed-frequency plaids was not drastically less efficient.

**Figure 3.**
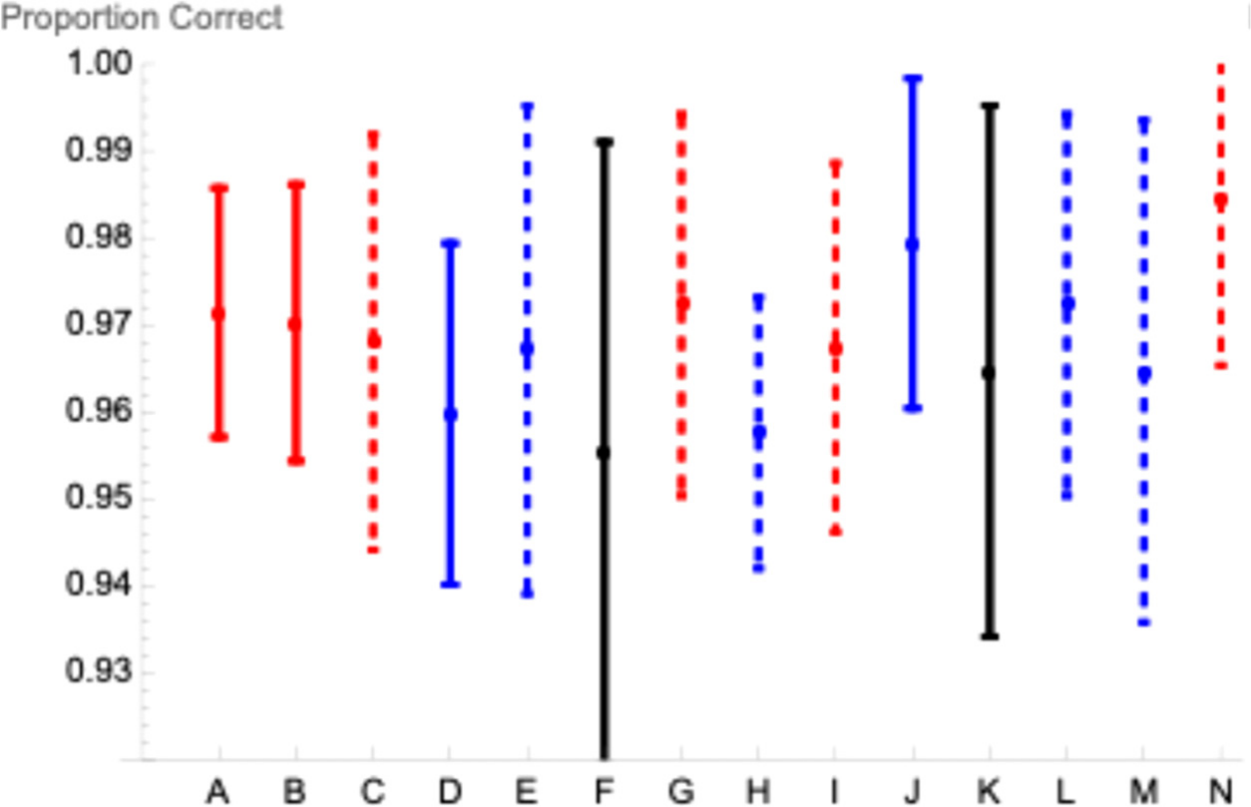
Weighted mean proportions correct ± 1 SD across participants for each of the 14 conditions indexed by the letters A–N in Figure 2. Color and dashing codes match those of Figure 2.

For each participant in each condition, response accuracy exceeded 90% correct. For each condition, weighted mean accuracy^3^ exceeded 95% correct. However, we note that mean accuracy was greatest (98.4% correct) in the condition N (in Figure 2) for which mean *RT* was longest (see Figure 3), raising the possibility of a small speed-accuracy tradeoff.

## Discussion

The data indicate that single-frequency plaids “pop out” from distractors identical to the components comprising it. As previously reported by Nam et al. (2009), average Δ*RT*/Δ*N* ratios were less than 5 ms/item. Furthermore, the data indicate search asymmetry: Δ*RT*/Δ*N* ratios increased (and thus, efficiency decreased) when participants attempted to locate isolated Gabors among single-frequency plaid distractors. Efficiency also decreased when participants attempted to locate isolated Gabors among mixed-frequency plaid distractors. What isn’t clear from the data is whether mixed-frequency plaids popped out from distractors identical to the components comprising them. The Δ*RT*/Δ*N* ratio for these plaids was larger than those for the single-frequency plaids, but nowhere near as large as the ratio (39.5 ms/item) reported by Nam et al. One possible reason for this difference is the greater similarity between low and high carrier frequencies used in this study. Another possible reason is the very different task (location) used here (Nam et al. used a yes/no detection task). Although seemingly remote, the possibility of individual differences among participants cannot be ruled out, either.

The search asymmetry for plaids implies that they contain a basic visual feature (i.e., one capable of guiding attention to a specific position in the visual field; Wolfe & Horowitz, 2004) not present in their component Gabors. This feature may be the “intrinsic two-dimensionality” that Barth et al. (1998) have argued is extracted from rectified Gaussian curvature in the visual intensity map.

Previous evidence for mechanisms preferring specific plaids to their component Gabors was described by Peirce and Taylor (2006), who compared two adaptation-induced reductions in apparent contrast. When the target plaid was identical to one of two plaids that were alternately exposed during adaptation, the reduction was greater than when neither of the adapting plaids matched the target, but each had one of the target's two components.

Besides preferring plaids to Gabors, little is known about the mechanisms mediating the efficient search for plaids. However, quite a bit has been discovered about the mechanisms mediating the appearance of plaids. Georgeson (1992; see also Meese & Freeman, 1995) has long argued that most plaids appear different from what would be expected on the basis of output from one-dimensional filters. Instead, their “checkerboard-like” appearance seems to be more consistent with zero-crossings in the output of an isotropic filter. On the other hand, Georgeson and Meese (1997) described observations inconsistent with that idea, too. They concluded in favor of an architecture that included “bridge” neurons preferring orientations between those of the plaid's components, as well as cross-orientation and cross-frequency interactions, plus something that detected the zero-crossings in filter output.

The mechanisms mediating plaid search may be the same as these mechanisms mediating the plaid appearance. On the other hand, it is also possible that they are different. For examples of visual targets that can be located with high efficiency despite their apparent similarity to distractors see Solomon et al. (2006) and Morgan and Solomon (2020).

## Figures and Tables

**Figure A1.**
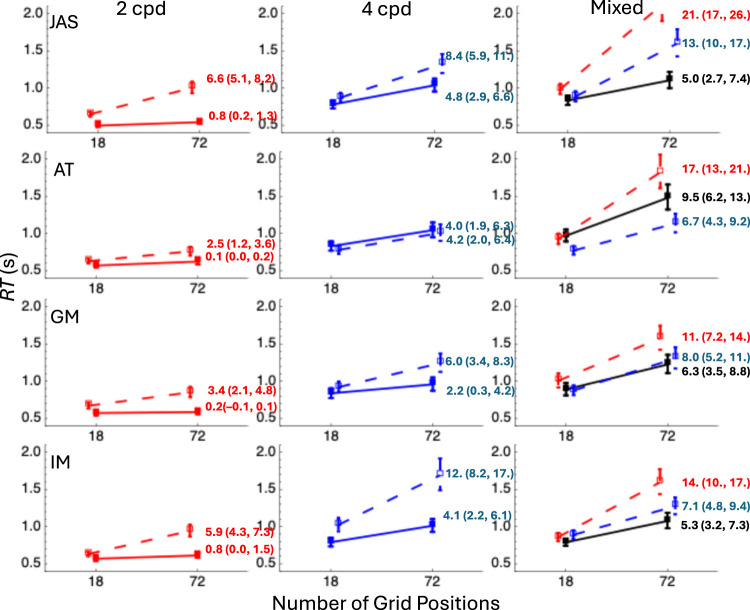
Geometric mean response times (*RT*) for each observer in each experimental condition. Error bars contain four standard errors of the mean. Color-coded numbers correspond to the ratio Δ*RT*/Δ*N* in ms/item. Red, blue, and black indicate 2-cpd, 4-cpd, and mixed-frequency targets, respectively. Parentheses contain Efron's (1987) 95% (BCα) bootstrap confidence interval.

## Notes

1. This is another notable difference between our methods and those of Nam et al. (2009), who used carriers having 2 and 5.25 cycles per degree. Informal observation suggested less of a visibility difference between 2- and 4-cycle-per-degree Gabors, especially when placed in the grid's corners. Empirical support for comparable visibility can be found in work by Georgeson and Shackleton (1993), who reported that 4-cycle-per-degree Gabors and plaids required no greater contrast to be judged equivalent with 2-cycle-per-degree Gabors and plaids having 32% Michelson contrast. (They did not report any results with greater values of contrast.) Their work also supports a √2 ratio, as two perpendicular Gabors formed a plaid whose contrast was judged equivalent with an isolated Gabor when the latter had approximately 3 dB more contrast.

2. Each participant's responses were given equal weight, even though some participants completed more trials than others.

3. We report mean accuracies rather than mean values of *d′* because some participants were 100% correct (*d′* = ∞) in some conditions.
